# Basal foraminifer endures anoxia via aerotolerant anaerobic mitochondria and unconventional energy metabolism

**DOI:** 10.1093/ismejo/wrag211

**Published:** 2026-08-13

**Authors:** Fatma Gomaa, Joan M Bernhard

**Affiliations:** Geology & Geophysics Department, Woods Hole Oceanographic Institution, Woods Hole, MA 02543 United States; Department of Organismic and Evolutionary Biology, Harvard University, Cambridge, MA 02138, United States; Geology & Geophysics Department, Woods Hole Oceanographic Institution, Woods Hole, MA 02543 United States

**Keywords:** anaerobic electron transport chain, aerotolerant mitochondria, anaerobic ATP generation, primitive eukaryotes, *in situ* preservation, Monothalamea, gene expression, (meta)transcriptome, Santa Barbara Basin

## Abstract

Deoxygenation is driving ecological shifts across vast oceanic volumes. To understand and predict future ocean ecosystem functioning and stability, it is critical to identify the eukaryotic metabolic repertoire that enables survival under anoxia. Foraminiferan protists are one of few eukaryotic lineages that can inhabit anoxic marine sediments, environments relevant today and in Earth’s past. Here, we investigate the metabolic strategies employed by a representative of an early-evolving foraminiferan group to persist anoxia. A saccamminid foraminifer inhabiting an anoxic bathyal seafloor in the Santa Barbara Basin (CA, USA) was preserved *in situ*. (Meta) transcriptomic analyses revealed an aerotolerant mitochondrial metabolism lacking Cytochrome *c* Oxidase, but expressing alternative oxidase, potentially fueled by internally released oxygen during reactive oxygen species detoxification, and utilizing a tricarboxylic acid reductive Complex II. This foraminifer uses glutamate oxidation and aspartate–malate shuttle to generate reducing equivalents, driving ATP production via an atypical anaerobic electron-transport chain. Energy metabolism is also tightly linked to phosphate availability, facilitating substrate-level phosphorylation of high-energy intermediates. This foraminifer’s competitive advantage is its anaerobic energy metabolism combined with ability to detoxify and reduce oxygen, if present. These unusual capabilities confer a blueprint for understanding how early eukaryotes may have evolved during anoxia, remained resilient during the Neoproterozoic Oxygenation Event, and likely will be ecological winners amid ongoing ocean deoxygenation.

## Introduction

The microbial eukaryote phylum Foraminifera, nearly ubiquitous in the marine realm, was historically considered canonically aerobic, but certain benthic foraminifera survive and even thrive in anoxia, performing diverse forms of anaerobic metabolism. To date, all foraminifers known to utilize anaerobic pathways, including nitrate respiration, have multichambered calcareous tests (shells) (i.e. belong to the Class Globothalamea), which evolved in the Carboniferous of the late Paleozoic, ~350 MYA, according to phylogenetic approaches [1]. In contrast, the metabolic capabilities of modern representatives of the foraminiferal class with the earliest radiation (Monothalamea) have received little study. Species belonging to this “basal” foraminiferal group have a single chamber (also known as “unilocular” forms) and the origin of the group is estimated to be between ~690–1100 MYA [1]. In addition to the phylogenetic estimates of Neoproterozoic origins of unilocular foraminifera, some Neoproterozoic microfossils have been considered foraminifera (i.e. vase-shaped microfossils of the Cryogenian and Ediacaran; [2, 3], respectively). Although debated, the Neoproterozoic ocean had much lower oxygen concentrations than those of present day [e.g. 4]. Thus, it is possible that ancestral unilocular foraminifera possessed oxygen-independent metabolic pathways.

**Figure 1:**
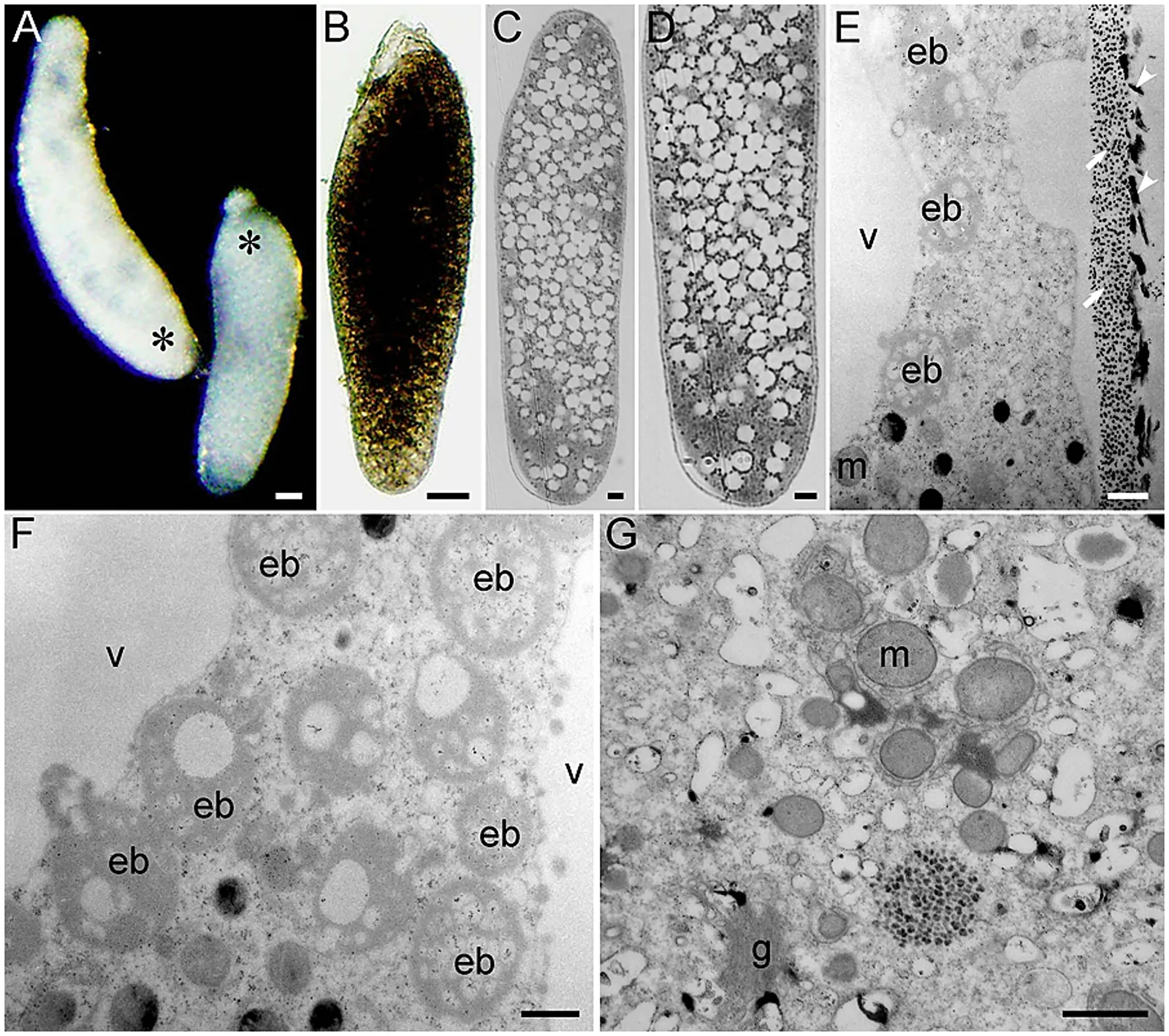
Micrographs of the Santa Barbara Basin saccamminid. (A-D) light micrographs. Reflected-light overview (A) of two specimens (one each with aperture up, down; * designates aperture end) showing white to cream coloring. Transmitted light view (B) showing large vacuoles. (C-D) transmitted-light micrographs of plastic-embedded specimen in longitudinal thick section, showing copious large vacuoles (C, D) and endobionts, dark entities occurring around vacuole periphery (D). (E-G) transmission electron micrographs. Layer of clay platelets (white arrowheads; E) overlying granulofibrillar test matrix (white arrows) and portion of vacuole (v) lined with endobionts (eb). (F) Endobionts associated with edge of vacuoles. (G) Area of cytoplasm lacking large vacuoles, showing mitochondria (m) and Golgi body (g). Scales: A = 25 μm; C-D = 10 μm; E-G = 0.5 μm.

There is at least one extant unilocular foraminifer species that inhabits anoxic, sulfidic sediments (Fig. 1) [5]. This taxon has putative endosymbionts [5] while having no apparent food vacuoles or peroxisomes [5, 6]. Such physiological observations are compelling considering recent emerging evidence of chemomixotrophy in the Globothalamea (calcareous multi-chambered) foraminifer *Nonionella stella* [7].

Copious populations of this endobiont-bearing unilocular foraminifer and the calcareous multi-chambered *N. stella* can be found in the deeper portions of the Santa Barbara Basin (SBB), located off southern California USA. This part of SBB typically is lowest in oxygen [8], highest in hydrogen sulfide [9], and often covered with white bacterial mat [7, 10]. Previous specimen counts indicated the SBB unilocular taxon was most abundant in the surface cm (~20 per cm^3^), but also in considerable abundance to 3-cm sediment depth (≥3.4 per cm^3^) [5], well below the penetration of dissolved oxygen, when present [e.g. 11]. The taxon has not been noted in well-aerated environments on the Basin’s flanks.

The SBB endobiont-bearing unilocular foraminifer is formally undescribed. Earlier publications [5, 6] referred to this species as an allogromiid (unilocular foraminifera lacking a mineralized test) because it appears to have an organic test. In reality, the SBB unilocular taxon has a very thin layer of clay platelets covering its thick organic test (Fig. 1E). Thus, the morphotype is technically a saccamminid (unilocular with an inorganic agglutinated test). In this contribution, we hereafter refer to this undescribed unilocular species as the “SBB saccamminid”.

Here, we report the gene expression of *in situ*-preserved populations of this SBB saccamminid from euxinic SBB sediments. These data provide insight into the metabolic role of an overlooked microbial eukaryote, the unilocular foraminifer, its contributions to modern biogeochemical cycling, and raise the possibility that similar metabolic strategies were employed by basal foraminifera in the Neoproterozoic.

## Materials and methods

### Sample collections

Foraminifera-bearing sediment samples were collected from ~571-m water depth at 34.29795, −120.06036 in the SBB (California, USA) in July 2021 using the Remotely Operated Vehicle (ROV) *Hercules*, as previously described [7]. The saccamminid specimens analyzed in this study were recovered from the same seafloor sediments that were preserved *in situ*—on the seafloor—to investigate gene expression in the kleptoplastidic foraminifer *Nonionella stella* [7]. Sediments were preserved *in situ* by injecting RNAlater into the head space of emplaced 8.2-cm diameter pushcores (Supplementary Fig. 2B and C in [7]). Immediately after RNAlater-preserved pushcores were available on surface support vessel E/V *Nautilus*, the surface ~2 cm of sediment was removed from each pushcore, RNAlater removed by rinsing with sterile 0.2-μm-filtered seawater (hereafter SFSW), and placed into clean HDPE bottles. *In-situ* preservation was confirmed due to presence of non-motile (preserved) nematodes.

### Sample preparation, ribonucleic acid extraction, and library preparation

All saccamminid pools presented in this study were isolated from sediments preserved *in situ,* on the seafloor, in RNAlater, as noted above. Seven small pools (n = 3, 4, 8, 12, 13, 13, 23) of the saccamminid were isolated—either at sea or in our laboratory—, cleaned with a fine brush, rinsed three times in clean SFSW, placed in RNase-free microfuge tubes with minimal SFSW and 100 μl of DNA/RNA Shield from ZymoResearch (USA). Four of these extracts, from pools of 3, 4, 8, 23 were used for total RNA library preparations (metatranscriptome; holobiont = host + symbionts). The three additional extracts, from pools of 12, 13, 13 were prepared for transcriptome library preparation (polyA to enrich for host mRNA).

Ashore, RNA was extracted from each pool using a Quick-RNA Microprep kit (Zymo Research). The extracted RNA was quantified with a Qubit Fluorometer, RNA quality was assessed using a Nanodrop, and RNA integrity was determined with a Bioanalyzer (Agilent 2100) and TapeStation (Agilent 2200), following manufacturer’s protocols. The total RNA yield per sample of pooled specimens ranged from 70 to 100 ng. RNA was then used to prepare libraries using two different kits: total RNA and poly (A)-enriched RNA library preparation kits, following our established methods [7, 12]. For metatranscriptome library preparation, a total RNA libraries were prepared using the Revelo RNA-Seq High Sensitivity library preparation kit (Tecan, USA), which employs proprietary SPIABoost technology to reduce unwanted ribosomal RNA and improve detection of low-abundance sequences. Additionally, we designed a custom probe specific for SBB saccamminid rRNA to bind and deplete ribosomal reads, thereby minimizing rRNA representation in the sequencing data. The cDNA was generated from total RNA (host + bacteria) using random and oligo (dT) primers. Libraries were then amplified using single-primer isothermal amplification (SPIA) and prepared with double-stranded cDNA fragmentation end repair to generate blunt ends and adaptor ligation. Libraries were then amplified and quantified by Bioanalyzer and TapeStation.

For transcriptomics data, the three remaining extracts were used to prepare enriched poly (A)-tailed mRNA libraries using oligo (dT) beads of Universal Plus mRNA-Seq library preparation kit with NuQuant (Tecan, USA). Each library, from total RNA and mRNA preparations, was assigned a unique eight-base barcodes. Libraries were pooled together and sequenced on one flow cell of a NovaSeq S4 System (Illumina), achieving ~150–250 million reads per sample (Bauer Core Facility, Harvard University).

### Data processing of ribonucleic acid reads

Reads were quality checked using TrimGalore (https://github.com/FelixKrueger/TrimGalore), which uses the adaptor trimming tools Cutadapt (https://cutadapt.readthedocs.io/en/stable/) and FastQC (www.bioinformatics.babraham.ac.uk/projects/fastqc/). Quality-controlled FASTQ files were concatenated into a single FASTQ file according to the type of library preparation, with three libraries from transcriptome data and four libraries from metatranscriptome data combined separately to enable co-assembly. De novo (meta)transcriptome assembly was conducted using Trinity v2.8.4 to generate contiguous transcript sequences (contigs). Transcript abundance for each sample was quantified with Salmon 0.12.0 [13]. Coding regions within the contigs were predicted using TransDecoder. The resulting sequences were then translated into amino acid sequences with the EMBOSS tool Transeq [14], which performs six-frame translations. This approach was used to ensure that no potential open reading frames were missed. Functional annotation of the amino acid sequences was performed using EggNOG-mapper v2, providing identifiers for KEGG (Kyoto Encyclopedia of Genes and Genomes) pathways and modules, as well as the NCBI Cluster of Orthologous Groups. Conserved protein domains were identified using the Subfamily Protein Architecture Labeling Engine in conjunction with the NCBI conserved domain database.

Transcripts were not binned using a dedicated binning pipeline. Instead, taxonomic origin was determined using sequence similarity–based approaches. Amino acid sequences were compared against reference databases using DIAMOND BLASTP and NCBI BLASTP; taxonomic assignment was inferred based on best-hit identity, alignment length, overall sequence similarity, and consistency with functional annotations derived from EggNOG and KEGG. Only proteins with at least 50% coverage against reference sequences were retained for downstream analyses. This approach allows discrimination between eukaryotic (foraminiferal) and bacterial sequences, including cases where bacterial mRNA may persist after poly(A) selection.

Because reference transcriptomic resources for foraminifera, and specifically for monothalamids, are extremely limited and no comprehensive transcriptome datasets are currently available, all sequences analyzed in this study were assembled de novo. Consequently, automated taxonomic assignment alone was insufficient to confidently assign sequences to foraminifera. All genes reported in the manuscript were, therefore, manually inspected on a gene-by-gene basis to confirm their taxonomic affiliation and to exclude obvious bacterial homologs. To identify bacterial sequences, we combined automated taxonomic annotation using EggNOG with manual curation. Sequences were classified as bacterial when BLAST searches returned predominantly bacterial hits (≥70–80% of the top matches) with high sequence identity (>40–50%).

**Figure 2:**
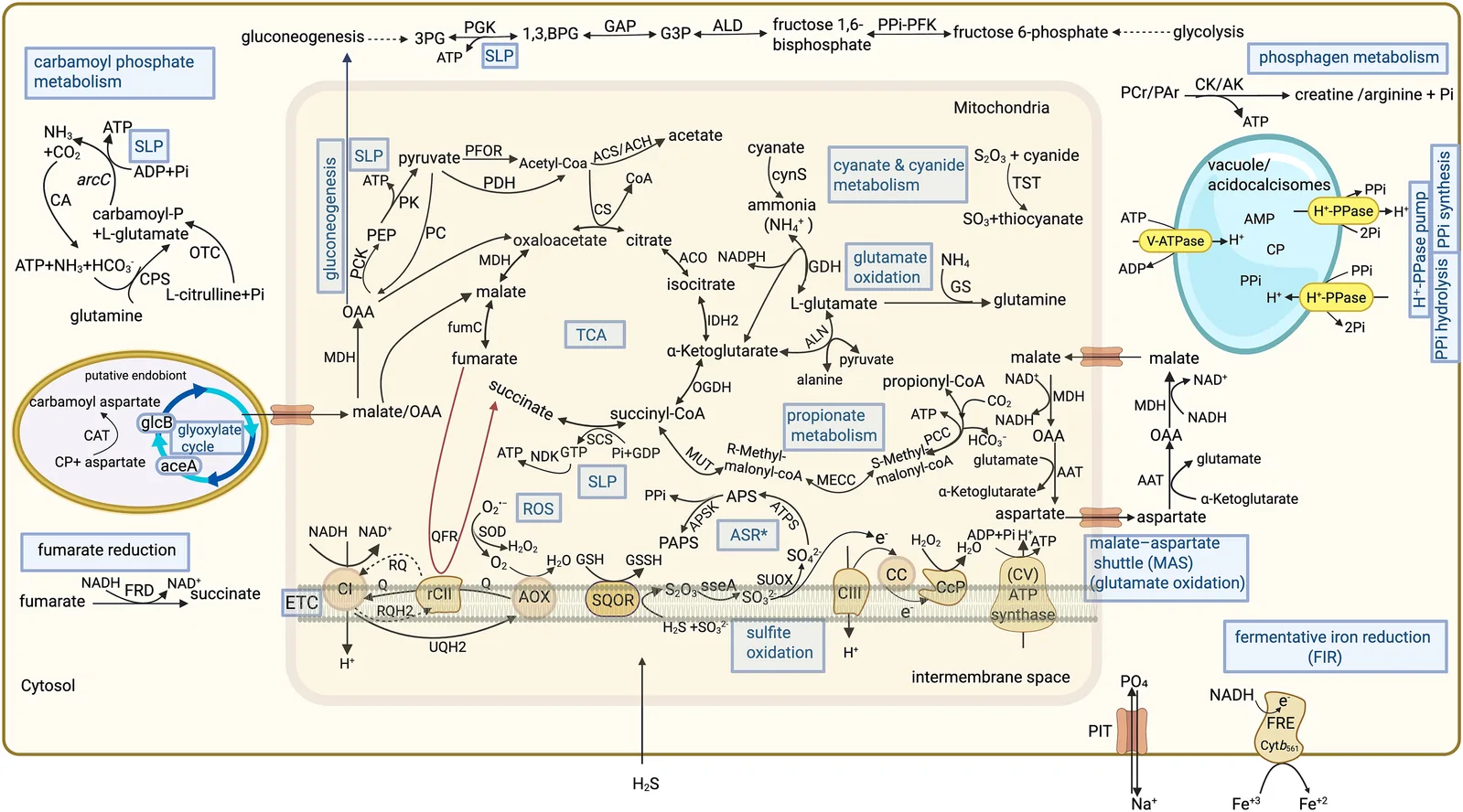
Schematic representation of key metabolic pathways in the SBB saccamminid and their putative *Pseudoalteromonas* endosymbiont based on gene expression data from the (meta)transcriptome reads. Biochemical conversions between metabolites are annotated with the abbreviation of corresponding genes, as defined in Supplementary Table 1. Identified proteins and metabolic pathways in the mitochondria are cyanate and cyanide metabolism; propionate metabolism; substrate-level phosphylartion (SLP); glutamate oxidation; mitochondrial branch of the malate-asparate shuttle (MAS); TCA cycle; gluconeogenesis; ROS detoxification; incomplete reductive sulfate assimilation (ASR*); sulfide oxidation (SUOX); mitochondrial electron transport chain components are: Complex I (CI); reductive complex II (rCII); alternative oxidase (AOX); sulfide quinone oxidoreductase (SQOR); complex III (CIII); cytochrome c (CC); cytochrome *c* peroxidase (CcP); ATP synthase (CV). Cytosolic metabolic pathways are CP metabolism; gluconeogenesis; phosphagen metabolism; fumarate reduction (NADH-FRD); cytosolic branch of the malate-asparate shuttle; and SLP. Metabolic pathways associated with the bacterial endobiont are glyoxolate cycle; carbamoyl aspartate metabolism. Vacuolar localized metabolisms are inorganic pyrophosphate hydrolysis and synthesis (H^+^-PPase pump). Cell membrane-associated metabolic pathways includes: Fermentative iron reduction (FIR) catalyzed by ferric reductase (FRE); inorganic phosphate transporter (PIT).

**Figure 3:**
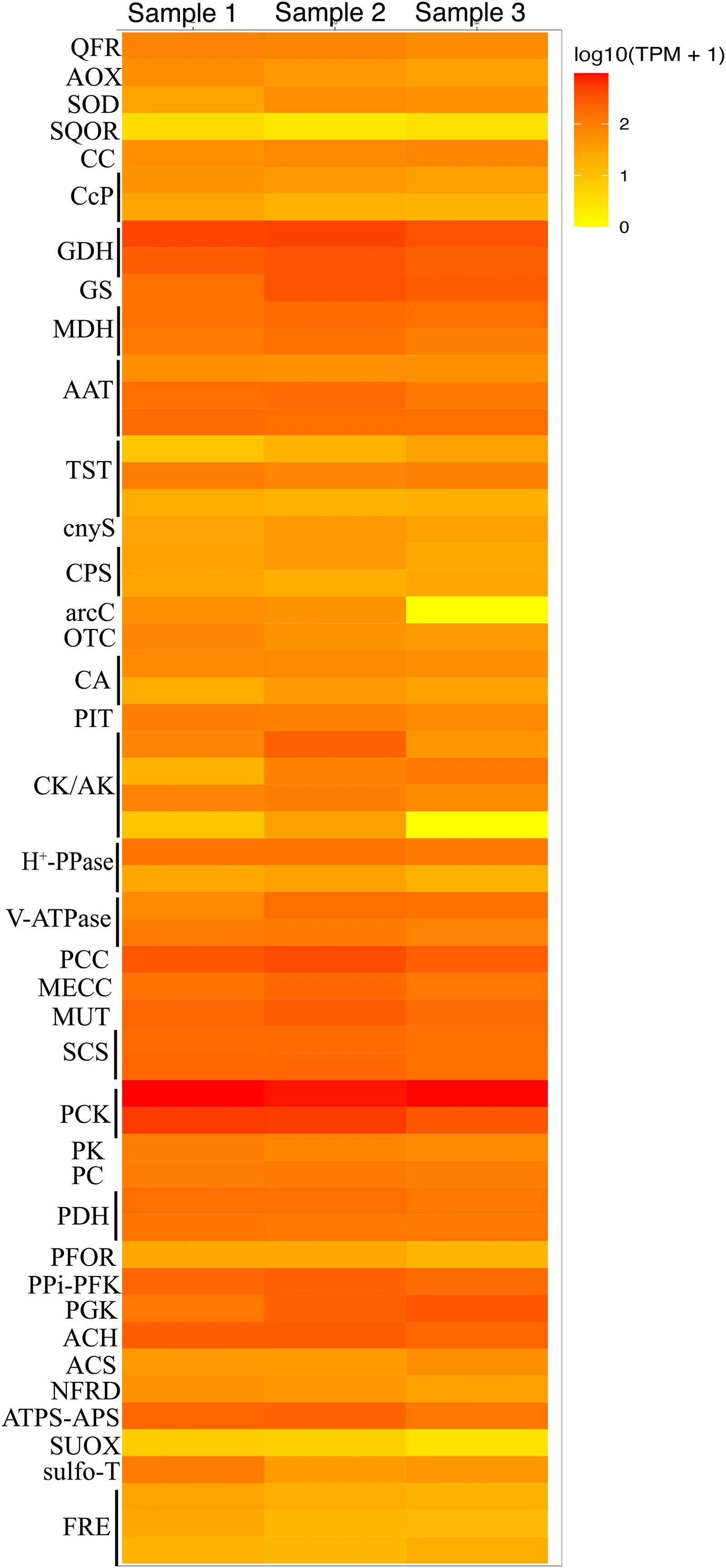
Heatmap showing the expression of transcripts retrieved from host (poly(A)-enriched) libraries associated with various metabolic pathways in the mitochondria and cytosol. Genes include rCII (quinol:Fumarate oxidoreductase, QFR), AOX (alternative oxidase), SOD (superoxide dismutase), SQOR (sulfide:Quinone oxidoreductase), CC (cytochrome C), CcP cytochrome *c* peroxidase (CcP), GDH (glutamate dehydrogenase), GS (glutamine synthetase), MDH (malate dehydrogenase), AAT (aspartate aminotransferase), TST (thiosulfate sulfurtransferase), *cynS* (cyanase), CPS (carbamoyl phosphate synthetase), *arcC* (carbamate kinase), OTC (ornithine transcarbamylase), CA (carbonic anhydrase), PIT (phosphate transporter), CK (creatine kinase), AK (arginine kinase), H^+^-PPase (membrane-bound proton-pumping pyrophosphatase), V-ATPase (vacuolar-type ATPase), PCC (propionyl-CoA carboxylase), MECC (methylmalonyl-CoA epimerase), MUT (methylmalonyl-CoA mutase), SCS (succinyl coenzyme a synthetase), PCK (phosphoenolpyruvate carboxykinase), PK (pyruvate kinase), PC (pyruvate carboxylase), PDH (pyruvate dehydrogenase), PFOR (pyruvate:Ferredoxin oxidoreductase), PPi-PFK (pyrophosphate-dependent 6-phosphofructokinase), PGK (phosphoglycerate kinase), ACH (acetyl-CoA hydrolase), ACS (acetyl-CoA synthetase), NFRD (NADH-fumarate reductase), ATPS-APS (bifunctional ATP-sulfurylase/adenylyl sulfate kinase), SUOX (sulfite oxidase), Sulfo-T (sulfotransferase), and FRE (ferric reductase).

Key genes were selected based on EggNOG annotations. Amino acid sequences were clustered at 95% identity using CD-HIT (Cluster Database at High Identity with Tolerance) to define representative sequences for each cluster. Metabolic maps of mitochondrial and cytosolic pathways were constructed using KEGG annotations and refined according to the literature. To visualize gene expression patterns across samples, heatmaps were generated from transcript-per-million (TPM) normalized transcript counts using ggplot2 v3.3.2 in R [15]. Amino acid and nucleotide sequences reported in the study (e.g. those shown in Figs 2 and 3), along with annotation are included in Supplementary Table 1.

### DNA extraction, library preparation, and metagenomes assembled genomes analysis

DNA was extracted from two independent pools of SBB saccamminid samples, each consisting of 25 cells, using the DNA Microprep Kit (Zymo Research). Libraries were prepared at the Harvard Bauer Core Facility using an Illumina DNA library preparation kit and sequenced on a NovaSeq S4 platform, generating ~312 million paired-end reads per sample. The reads were assembled using MEGAHIT [16], followed by gene prediction on the assembled bins. Predicted proteins were functionally annotated using eggNOG, and mitochondrial localization was assessed computationally. Gene sequences were exported as FASTA files for downstream homology-based analyses, such as BLAST. To minimize redundancy, sequences were clustered at 100% nucleotide identity (Supplementary Data 1 and 2).

### Organelle localization predictions

Mitochondria or cytosol localizations were predicted using PredSL and TargetP version 2.0. Localization predictions of PredSL were based on homology using Markov Chains and a hidden Markov model for the prediction of the subcellular localization of proteins in eukaryotic cells from the N-terminal amino acid sequence, whereas localization predictions using TargetP detect and identify the types of N-terminals targeting peptides (e.g. mitochondrial targeting peptides and signal peptides) to infer protein localization [17, 18]. Sequences were considered to contain a signal peptide or mitochondrial targeting peptide if their scores exceeded a probability threshold of 0.5 in either TargetP or PredSL (Supplementary Table 1), following the criteria used in Powers et al. [19], which reported results for two benthic foraminiferal species, *Bolivina argentea* and *Nonionella stella.*

## Results and discussion

The SBB saccamminid has evolved specialized mitochondrial metabolisms to survive in their anoxic, highly sulfidic environment (Table 1). While transcripts support a functional electron transport chain (ETC) and tricarboxylic acid (TCA) cycle, expression of Cytochrome *c* Oxidase (CcO), a hallmark for aerobic respiration, was not noted in any sample (n = 7; Fig. 2). Transcriptomic data reveal that the ETC consists of Complex I (CI), reductive Complex II (rCII), Complex III (CIII), and Complex V (CV; ATP synthase) (Fig. 2; Table 1; Supplementary Data 3 and 4). CcO subunits that are nuclear- or mitochondrial-encoded were not recovered in the metatranscriptomic or MAG analyses; the lack of recovery of mitochondrial-encoded CcO subunits may reflect incomplete assembly or recovery of mitochondrial-encoded CcO genes, as the mitochondrial genome composition of this group remains unknown. In contrast, the lack of recovery of nuclear-encoded CcO subunits from our metatranscriptomic and MAG datasets provides additional support for the inference that CcO was not expressed under the *in situ* conditions, particularly because we detected expression of other nuclear-encoded, mitochondrial-targeted genes involved in the ETC, TCA cycle, and ATP synthesis (assembled contigs are provided in Supplementary Data 1 and 2). Instead, alternative oxidase (AOX) transcripts were consistently detected across all samples, in both transcriptomic (host) and metatranscriptomic (host + associated endobiont) datasets.

**Table 1:** Metabolic pathways performed by SBB saccamminid.

**Metabolic pathway**	**Description / Function**	**Occurrence**
TCA cycle with reductive CII	Complete (rCII, fumarate as a terminal electron acceptor) with QFR replacing SDH	Anaerobic microeukaryotes & certain foraminifera (*N. stella*)
ETC lacking CIV (lacks Cytochrome *c* Oxidase)	CI, rCII, CIII, and ATP synthase expressed; supports modified anaerobic ETC	Protists with reduced or absent mitochondria (i.e. MROs); new to foraminifera (This study)
Alternative ETC components	AOX terminal oxidases use O_2_ produced intracellularly by SOD and SQOR for H₂S oxidation and detoxification	Plants, microeukaryotes including foraminifera from hypoxic/anoxic, sulfidic habitats
Glutamate oxidation	Glutamate dehydrogenase and malate–aspartate shuttle oxidize glutamate, providing TCA intermediates and fueling mitochondrial electron flow for ATP generation	Prevalent in eukaryotes but here likely supports anaerobic energy metabolism in this foraminifer lacking C*c*O (This study)
Aspartate–malate shuttle	Transfers reducing equivalents from cytosol to mitochondria, supporting redox balance by maintaining ATP production & recycling NAD^+^ under low- O₂ conditions	Widespread in eukaryotes; likely supports anaerobic energy metabolism in SBB saccamminid (This study)
Substrate-level phosphorylation	Generates ATP by transferring a phosphate group from high-energy substrates to ADP molecule	Certain anaerobic eukaryotes including some foraminifera; supports anaerobic energy metabolism
Cyanide/cyanate metabolism	Cyanase catalyzes the production of ammonium, providing a nitrogen source, while TST (rhodanese) detoxifies cyanide and participates in sulfur metabolism	Marine microeukaryotes; cyanase new to foraminifera (This study)
Carbamoyl phosphate metabolism	Intracellular Pi stimulates energy production from carbamoyl phosphate during anaerobiosis	Protists with reduced or absent mitochondria (i.e. MROs); new to foraminifera (This study)
Glyoxylate cycle	Occurs in putative endobiont but inconsistent expression suggests plasticity of bacterial association	Host associated in plants and microeukaryotes including some foraminifera (*N. stella*)
H^+^-PPase pump & phosphogen metabolism	During anoxia, cells use energy-rich compounds (e.g. creatine/arginine phosphate); polyphosphates, likely stored in putative acidocalcisomes, also help control pH	Plants and microeukaryotes including some foraminifera
Propionate metabolism	Supports ATP production during anoxia, activated by intracellular Pi, & relies on V-PPase to maintain pH	Certain eukaryotes; new to foraminifera (This study)
Gluconeogenesis	During anoxia, glucose is produced from amino acids to support energy metabolism when external C sources and O_2_ are limited; no evidence of phagocytosis in this foraminifer	Eukaryotes including foraminifera
Assimilatory sulfate reduction (partial)	Incorporates sulfate into organic compounds	Certain eukaryotes including foraminifera
Fermentative iron reduction (FIR)	Uses Fe^+3^ as alternative electron sink, transferring reducing equivalents from fermentable substrates to Fe^+3^, which is reduced to Fe^+2^	Many fungi and microeukaryotes; new to foraminifera (This study) suggesting a role in local iron cycling under anoxic conditions

We also detected expression of Cytochrome *c* Peroxidase (CcP), which oxidizes reduced Cytochrome *c* using hydrogen peroxide (H₂O₂) as an electron acceptor to produce water (Figs 2 and 3). However, CcP functions in peroxide detoxification and does not replace Complex IV in respiratory electron transport. In contrast, AOX reoxidizes the quinone pool by transferring electrons from reduced quinol directly to oxygen, which acts as a terminal electron acceptor, but lacks proton-pumping ability across the mitochondrial membrane [20]. AOX is commonly found in organisms that face high concentrations of hydrogen sulfide (an inhibitor of aerobic respiration) and those adapted to anoxic environments, including certain fungi, protists, and metazoans [20]. Together, CcP and AOX likely help maintain electron flow under low-oxygen conditions, providing alternative respiratory routes that limit proton motive force generation compared to the canonical pathway.

Given that the SBB saccamminid inhabits euxinic sediments, it may rely on reactive oxygen species (ROS)-scavenging systems to manage oxidative stress. This is supported by the consistent expression of mitochondria-localized superoxide dismutase (SOD) across all transcriptomes (Fig. 3) and metatranscriptomes. We did not detect the expression of catalase, an enzyme that reduces hydrogen peroxide, in any of our sequence data. These findings support previous TEM imaging studies, which noted the absence of peroxisomes in this morphotype [5]. Instead, this foraminifer expresses heme peroxidase, presumably to manage H₂O₂ toxicity. We, therefore, suggest that the SBB saccamminid is an aerotolerant anaerobe —a microeukaryote that lives permanently in anoxic conditions but can survive limited exposure to oxygen. Some archaeal species exhibit similar strategies, expressing superoxide dismutase (SOD) but lacking catalase [21]. Certain metazoan species tolerate sulfide-rich environments by expressing sulfide quinone oxidoreductase (SQOR), an enzyme that plays a key role in oxidizing hydrogen sulfide for both detoxification and energy metabolism [22, 23]. Low expression of SQOR was detected in all SBB saccamminid samples (Fig. 3), suggesting that sulfide oxidation is unlikely to be the main electron donor pathway in this foraminifer, although it may still contribute to its respiratory chain. SQOR has also been detected in Globothalamids from low oxygen to euxinic settings, *B. argentea* and *N. stella* [7, 12, 19].

All TCA-cycle enzymes were expressed, including transcripts encoding succinate dehydrogenase (SDH; Complex II), which are orthologous to the canonical Complex II found in other aerobic eukaryotes. However, these sequences do not appear to function as the canonical oxidative form of Complex II; instead, they contain conserved features characteristic of quinol:fumarate oxidoreductase (QFR), including a diagnostic glycine residue in the quinone-binding site that enables rhodoquinone binding [19, 24]. Consistent with this, we also detected the rhodoquinone-biosynthesis methyltransferase, which, together with these QFR-like features, supports our inference that Complex II may operate in the reductive direction (rCII) (Figs 2 and 3). This arrangement could bypass the Q-cycle, thereby limiting Complex III activity, which in this context cannot function as a proton-pumping complex. This inferred fumarate-reducing activity of Complex II is consistent with observations in other SBB foraminifera such as *N. stella* [19], and with anaerobic mitochondrial metabolism in which fumarate is the terminal electron acceptor to generate succinate [23, 25–27]. While reversal of Complex II in hypoxic human mitochondria has been primarily associated with transient ROS production [28], recent evidence suggests that rhodoquinone (RQ) may enable sustained electron transfer to fumarate via reversed succinate dehydrogenase activity, even in the absence of O₂ [29]. The expression of both QFR and AOX in our (meta)transcriptomes suggests that these enzymes play a role in re-oxidizing the rhodoquinone (RQH₂) and ubiquinol (UQH_2_) pools, acting as electron carriers, indicating that this foraminifer may partition electron flow and can use two different electron acceptors (fumarate and oxygen) simultaneously. Additionally, soluble fumarate reductase (FRD-NADH), an enzyme previously identified from foraminifera-rich anoxic sediments [30], was expressed in our samples.

We interpret our findings—specifically the expression of AOX with the absence of CcO expression—in the context of a prior study on reticulopodial motility in two allogromiid foraminifera [31], close relatives of saccamminids [32]. That study [31] demonstrated that neither cyanide (a CcO inhibitor) nor an AOX inhibitor alone was sufficient to halt cellular activity. However, when both inhibitors were applied simultaneously, motility ceased entirely, suggesting that both terminal oxidases were present and functionally redundant in those allogromids. In contrast, the SBB saccamminid expresses AOX, whereas transcripts corresponding to CcO were not detected in our data. This observation suggests that AOX-based respiration may permit continued electron flow under cyanide exposure and prompted us to consider additional pathways for energy metabolism, redox balance, and potential detoxification of cyanide or its derivatives.

One of the most expressed transcripts in our data is glutamate dehydrogenase (GDH) (Fig. 3), a glutamate oxidation enzyme that drives the formation of α-ketoglutarate from glutamate to generate reducing powers (electrons; NADPH/NADH). The α-ketoglutarate could be further utilized to generate succinyl-CoA for substrate-level phosphorylation (SLP), a route that requires inorganic phosphate (Pi) to support energy metabolism. α-ketoglutarate can also be used in the TCA cycle reductive branch to increase the fumarate pool through reductive carboxylation by NADP^+^-dependent isocitrate dehydrogenase (IDH2) (Figs 2 and 3). We also detected expression of the nucleotide transhydrogenase (NNT), a mitochondrial inner-membrane protein and major source of NADPH, across all samples. Similar metabolic rerouting has been reported in mammalian mitochondria when aerobic respiration is suppressed, where bidirectional flux through oxidative α-ketoglutarate metabolism generates the reducing power (NADH) needed to enable reductive carboxylation [33–35]. This NADH serves as substrate for NNT-mediated NADPH production, with IDH2 acting as a primary consumer of NADPH during the reverse reaction [33, 35]. Together, these findings support the possibility that this foraminifer employs both oxidative and reductive branches of α-ketoglutarate metabolism to sustain NADPH-dependent reverse IDH2 activity and maintain fumarate production for respiration via QFR (Fig. 3).

An additional route for glutamate oxidation uses malate dehydrogenase (MDH) in the malate–aspartate shuttle (MAS) that involves both mitochondrial aspartate aminotransferase (AAT), also known as glutamate oxaloacetate transaminase, and its cytosolic counterpart (Fig. 2). Both MDH and AAT were highly expressed in all transcriptomes (Fig. 3) and metatranscriptomes. The reducing equivalents (NADH) produced from glutamate oxidation can enter the ETC, ultimately enhancing ATP production [36]. The glutamate oxidation pathway, therefore, appears to play an essential role in supporting mitochondrial ATP production in this foraminifer. Up-regulation of MDH, AAT, and QFR expression was observed in *N. stella* incubated under anoxic conditions supplemented with NO₃^−^ [19], suggesting that Globothalamea also possess the genetic capacity to operate the malate–aspartate shuttle.

Pathways associated with cyanide and cyanate detoxification were detected in our data. The expression of thiosulfate:cyanide sulfurtransferase (TST), which converts cyanide into thiocyanate [37], was noted in all samples. Additionally, cyanase (*cynS*), a gene that plays a key role in nitrogen metabolism in many marine microeukaryotes by facilitating conversion of cyanate into ammonia via a bicarbonate-dependent reaction [38], was consistently expressed across all transcriptomic (Figs 2 and 3) and metatranscriptomic data, with localization predictions indicating its presence in mitochondria. This finding suggests that the SBB saccamminid may use cyanate as a nitrogen source to produce ammonium (NH_4_^+^) that can be converted to glutamate via GDH, and subsequently to glutamine through the action of glutamine synthetase (GS). The resulting glutamine serves as a key metabolic intermediate, as it can be further oxidized to augment the production of reducing power for mitochondrial energy metabolism (as noted above). Cyanate, although present at low concentrations in seawater [39, 40], can be generated through the oxidation of cyanide and the decomposition of urea, as well as through the degradation of carbamoyl phosphate (CP), a high-energy compound commonly found in bacterial species inhabiting anoxic environments [38, 41, 42].

To investigate potential internal sources of cyanate, we searched for genes involved in CP metabolism. Carbamoyl phosphate synthetase (CPS), which catalyzes the formation of CP and glutamate from bicarbonate, ammonium and glutamine, was expressed in all samples and encoded in the foraminifer with cytosolic localization (Figs 2 and 3). An alternative pathway for CP generation, which involves citrulline via catabolic ornithine transcarbamylase (OTC) [42], was also expressed. Carbamoyl-p is a high-energy compound that can be used for ATP generation via carbamate kinase (*arcC*) [43], also highly expressed in our data (Fig. 3). *arcC* has been identified in eukaryotes with anaerobic mitochondria-related organelles (MRO) [44]. Further, *arcC* is involved in the ATP-generating process of arginine degradation via the arginine deaminase pathway [44]. Carbamoyl phosphate can also be converted to carbamoyl aspartate via aspartate carbamoyl transferase (CAT), a reaction that plays a key role in preventing the accumulation of cyanate. Two isoforms of *pyrB*, the gene encoding ATCase (aspartate carbamoyltransferase), have similar expression levels and were detected exclusively in the metatranscriptome data (endobiont) (Supplementary Fig. 1). One isoform showed 100% amino acid identity with *Pseudoalteromonas marina*, while the other shared 70% similarity with *Bathyarchaeota*. These findings suggest a potential symbiosis between the saccamminid and *Pseudoalteromonas* and/or *Bathyarchaeota*. This association likely coordinates the CP metabolism between the foraminifer host and endobiont(s) to prevent cyanate toxicity. In bacteria, previous studies have shown that maintaining a steady concentration of both CPS and CAT is crucial to avoid cyanate buildup plus associated toxicity [45]. Therefore, the expression of *pyrB* (in the bacterial endobiont) and cyanase (*cynS*, in host mitochondria) in the SBB saccamminid is likely for detoxification of internally produced cyanate from CP degradation.

We systematically examined the metatranscriptome data to evaluate both the taxonomic diversity and potential functional contributions of the bacteria associated with the SBB saccamminid. We identified ~10 bacterial groups that each contribute >1% of total TPM. The most abundant groups include Gammaproteobacteria, Actinobacteria, Rhodospirillales, and an unclassified bacterial lineage, with relative abundances varying among samples (Supplementary Fig. 2). These analyses demonstrate that the SBB saccamminid can host a diverse bacterial assemblage that differs across samples. Although Gammaproteobacteria (including *Pseudoalteromonas*) were prominent in some samples, expression of two glyoxylate cycle genes (*aceA* and *glcB*) with sequence identity to *Pseudoalteromonas* was detected at high levels in only one of the four samples, with the remaining samples showing little to no expression of these genes. Given this pattern, we cannot determine the stability or specificity of any individual bacterial group in the host-associated community. This pattern suggests that *Pseudoalteromonas* may contribute to carbon metabolism within the host-associated community, but that this activity may vary across individuals/ populations and/or over time. TEM images show that the morphology and size of the endobiont can differ from those documented in previous work (Supplementary Fig. 3) [5]. These findings further support the possibility of symbiotic plasticity in the SBB saccamminid. The expression of glyoxylate-cycle genes in the endobiont does not necessarily imply that the resulting metabolites are transferred between partners. However, it raises the possibility that under certain conditions, these byproducts could contribute to host–symbiont carbon flux if metabolite exchange occurs.

In our transcriptome and metatranscriptome datasets, we detected no expression of denitrification-related genes in any samples, contrasting prior findings of nirK in the pseudomonad endobiont [6]. This observation aligns with studies showing that closely related unilocular species exhibit variable nitrate storage and denitrification activity among individuals [46]. Overall, symbiont and microbiome specificity remain poorly understood in this diverse protistan group.

This SBB saccamminid foraminifer possesses the metabolic capability to generate ATP through cytosolic phosphagen (guanidino) kinases such as creatin kinase (CK) and arginine kinase (AK), in agreement with Orsi et al. [30]. Two isoforms of phosphagen kinases were highly expressed in all SBB saccamminid (meta)transcriptomes, both with cytosolic localization (Fig. 2). These enzymes catalyze the generation of ATP from the high-energy storage compounds phosphocreatine and phosphoarginine. Phosphagen kinase expression levels increase during anaerobic metabolism and these enzymes regulate the intracellular levels of inorganic phosphate (Pi) [47]. The SBB saccamminid expresses an inorganic pyrophosphate (PPi)-driven H^+^-PPase and an ATP-dependent V-ATPase. Together, these proton pumps contribute to establishing the H^+^ electrochemical potential across the vacuolar membrane. H^+^-PPases have been described in the calcareous foraminifer *N. stella* [7]. In plants and other protists, the pyrophosphate-dependent enzyme pumps protons from the cytosol into the vacuole compartments (e.g. acidocalcisomes) contributing to organelle acidification and pH homeostasis, particularly under hypoxic or energy-limited conditions that promote cytosolic acidification [48, 49]. However, it is unclear whether the two pumps coexist in the same vacuole in this SBB saccamminid. Storage of high-energy phosphate compounds such as creatine phosphate, pyrophosphate, and polyphosphate in the vacuoles of certain foraminifera species has recently been recognized as a possible adaptive mechanism for maintaining an energy source when electron acceptors are depleted under low-O_2_ conditions [50]. Our data supports the idea that SBB saccamminid actively utilizes a PPi-dependent metabolic pathway (Fig. 2). This is evidenced by the expression of key pyrophosphate-dependent enzymes, including 6-phosphofructokinase (PPi-PFK) and H^+^-PPase (Fig. 3). From a structural perspective, this foraminifer clearly has a plethora of large “empty” vacuoles (Fig. 1C–F) [5, 6] that would serve this capacity to use (pyro)phosphate well. Future research investigating gene expression under phosphate-limited or phosphate-starved conditions may provide further insights into the role of phosphate in the energy metabolism of benthic foraminifera.

Propionate metabolism likely plays a crucial role in energy conservation in this SBB saccamminid. The three components of this reversible metabolic pathway— Propionyl-CoA carboxylase (PCC), methylmalonyl-CoA mutase (MUT), and methylmalonyl-CoA epimerase (MECC)— were all strongly expressed (Fig. 3). Depending on the pathway direction, the end product is either propionate, a low-energy compound, or succinyl-CoA/succinate, an energy-rich intermediate that can further contribute to ATP production via succinyl-CoA synthetase (Fig. 2). PCC catalyzes the reversible conversion of (S)-methylmalonyl-CoA to generate propionyl-CoA, ATP, and bicarbonate. This pathway, which has been reported in protists and metazoans adapted to hypoxic and anoxic habitats. PCC activity is sensitive to the cellular energy status and is enhanced when ADP and inorganic phosphate levels are elevated under low-energy conditions. PCC is also pH-sensitive: mildly acidic conditions (i.e. lower mitochondrial-matrix pH during anaerobiosis) stimulate its activity [51–53]. PCC activity contributes to intracellular acid–base homeostasis by producing bicarbonate, which buffers acidosis and helps stabilize pH during anaerobic metabolism.

Gluconeogenesis is a central pathway in this foraminifer, similar to observations in *N. stella* [7]. High expression of mitochondrial phosphoenolpyruvate carboxykinase (PCK) and detectable expression of pyruvate carboxylase (PC) were noted in all samples (Fig. 3). Together, these two enzymes regulate the production of oxaloacetate (OAA), a key precursor for gluconeogenesis. Other enzymes associated with pyruvate metabolism, including pyruvate dehydrogenase (PDH) and pyruvate kinase (PK), were also expressed in all samples.

Three isoforms of ferric reductase (FRE), a plasma-membrane–associated oxidoreductase that converts insoluble Fe (III) to soluble ferrous iron (Fe (II)) using NADH or NADPH as an electron donor, were expressed (Fig. 3). Microbial iron reduction is a major process in anoxic marine sediments [54, 55]. Ferric reduction is typically classified into three categories: (a) assimilatory iron reduction, where ferric reductases mediate the uptake of iron from the extracellular environment for incorporation into intracellular proteins; (b) dissimilatory iron reduction, in which the oxidation of organic matter is coupled to iron reduction for energy conservation [56]; and (c) fermentative iron reduction (FIR), where reducing equivalents generated during the catabolism of fermentable substrates (e.g. pyruvate) are transferred to Fe(III), which thereby serves as an electron sink [55]. We suggest that the FRE isoforms identified in this SBB saccamminid function primarily as a FIR, serving as an alternative electron sink for cytosolic NADH/NADPH. Thus, FRE-mediated reduction is critical for sustaining redox balance and indirectly enriching Fe^2+^ in the surrounding environment (Fig. 2). The released Fe^2+^ could be utilized by Fe (II)-oxidizing microbes, including those that perform nitrate-reducing Fe (II) oxidation during anoxic conditions [57]. Future studies are needed to determine how widespread iron metabolism is in foraminifera and to elucidate the ecological role of saccamminids in local iron cycling.

ATP-sulfurylase-adenylyl sulfate kinase (ATPS-APS kinase), a bifunctional enzyme involved in the first step of assimilatory sulfate reduction (ASR) identified in *N. stella* [7], was expressed in our saccamminid data (Fig. 3). Because no sulfite-reduction genes were detected (Figs 2 and 3), our results suggest that these two SBB foraminifera cannot perform complete ASR. Instead, they form phosphoadenosine phosphosulfate that can be used for sulfation of proteins and lipids, via sulfotransferases (Sulfo-T), which are highly expressed in our data (Fig. 3).

Foraminiferal viability was confirmed by the expression of key genes related to their cellular cytoskeleton, growth, and metabolism, as well as protein synthesis and transport (Supplementary Fig. 4). These data indicate that these foraminifera were metabolically active at the time of sea-floor preservation, and not in a dormant or encysted state.

In sum, our study reveals that this monothalamid foraminifer employs QFR as a terminal oxidase for fumarate reduction and possesses AOX to maintain electron flow across the ETC—yet notably lacks Cytochrome *c* Oxidase (CcO), the canonical hallmark of aerobic respiration. Despite this, it maintains a complete TCA cycle, including a reductive branch (rCII), indicating an atypical hybrid respiration. Additionally, our study shows that both mitochondrial and cytosolic metabolisms are tightly coupled to inorganic phosphate for energy conservation through substrate-level phosphorylation of high-energy compounds. This trait may reflect ancient constraints on bioenergetics prior to the widespread use of canonical oxidative phosphorylation. From an evolutionary perspective, the SBB saccamminid appears to preserve a metabolic architecture that is distinct from that described for Globothalamea, as indicated by the lack of Cytochrome *c* Oxidase and the expression of oxygen-detoxification pathways (e.g. AOX and SOD) that detoxify intracellularly produced oxygen. These features are consistent with metabolic traits that may have been advantageous in low-oxygen environments such as those characteristic of Neoproterozoic deep oceans, prior to the widespread emergence of fully aerobic respiration. However, we cannot exclude the possibility that some of these metabolic characteristics represent secondary adaptations to persistent anoxia, similar to certain species of ciliates. Increased species sampling across Monothalamous (unilocular) foraminifera will be essential to distinguish between ancestral metabolic retention and secondary adaptation. In sum, the reported metabolic strategies in the SBB saccamminid highlight the remarkable plasticity of foraminiferal energy conservation pathways, providing valuable insight into eukaryotic metabolism and its role in the marine biogeochemical cycling and ecosystem resilience under deoxygenated conditions.

## Supplementary Material

Supplementary_material_wrag211

## Data Availability

All data presented in this manuscript are submitted to the NCBI under the BioProject submission number PRJNA1419926. The raw transcriptome and DNA sequencing reads are deposited in the NCBI repository SUB15985312 and SUB16095075. Additionally, the final processed files, including each transcriptome’s assembled sequences, predicted functions, and sample-specific abundances, have been uploaded to Figshare (https://doi.org/10.6084/m9.figshare.30287110). Additional data or codes related to this paper may be requested from the authors.
