# Supplementary material for: Basal foraminifer endures anoxia via aerotolerant anaerobic mitochondria and unconventional energy metabolism
Source: ISME J. 2026 Aug 13;20(1):wrag211. doi: 10.1093/ismejo/wrag211 (PMC13561418; doi:10.1093/ismejo/wrag211)
Supplement: Supplementary file 1 [file supplementary_material_wrag211.zip › Supplementary Figure legends.docx]

**Supplementary Figure legends:**

**Supplementary Figure 1.** Heatmap showing gene expression from total RNA libraries (metatranscriptomic data), highlighting genes associated with the putative *Pseudoalteromonas* endobiont. These are *aceA* (isocitrate lyase) and *glcB* (malate synthase), both key enzymes of the glyoxylate cycle; and *pyrB* (aspartate carbamoyltransferase) and CPS (carbamoyl phosphate synthetase), which are involved in carbamoyl metabolism. Detailed analyses showing the bacterial diversity associated with the SBB saccamminid are shown in Supplementary Fig. 2.

**Supplementary Figure 2.** Metatranscriptomic analysis of four SBB saccamminid samples showing the composition and relative abundance of associated bacteria: (A) Taxonomic composition of the metatranscriptome, showing the relative contribution of major bacterial groups and eukaryotic transcripts, expressed as the percentage of total transcripts per million (TPM); (B) Total TPM percentage contributed by each bacterial and eukaryotic group across the four samples, illustrating sample-specific variation in community composition; (C–F) Distribution of functional annotations based on TPM counts for genes assigned to different bacterial groups, highlighting the most highly expressed bacterial functions. Across samples, approximately ten bacterial groups each contribute >1% of total TPM; panels C–F highlight four representative groups, (C) Gammaproteobacteria (including *Pseudoalteromonas*), (D) Actinobacteria, (E) Rhodospirillales, and (F) an unclassified bacterial lineage, among the most abundant. Taxonomic assignments are based on sequence similarity to reference databases. Sequences labeled as, for example, ‘Metazoa’ represent the closest match in the database and do not indicate the presence of metazoan contamination in the sample.

**Supplementary Figure 3**. Transmission Electron Microscopy (TEM) images of endobionts in the SBB saccamminid: (A-B) Endobionts (arrows) with scalloped edges and coccoid shape.  These differ in shape and size from those described in previous studies (Bernhard et al 2006; 2012). This morphological variability suggests symbiotic plasticity within the SBB saccamminid host. Scales = 0.5 µm.

**Supplementary Figure 4.** Heatmap presenting the expression values of key housekeeping genes related to cell growth, metabolism, protein synthesis, and transport, including Actin, Tubulin, Microtubule-associated proteins (MAP), Dynein light chain, Myosin, 60S and 40S ribosomal proteins, Glyceraldehyde-3-phosphate dehydrogenase (GAPDH), and GTP-binding nuclear protein—a GTPase involved in nucleocytoplasmic transport.

**Supplementary Data legends:**

**Supplementary Table 1.** List of genes reported in this study (Figs. 2 and 3), including their amino acid and nucleotide sequences, functional annotations, and mitochondrial localization prediction scores (e.g., TargetP and PredSL).

**Supplementary Data 1.** The Contig column represents gene identifiers retrieved from the metagenomic assembly; TargetP Probability and PredSL Probability, are the predicted probability of mitochondrial localization based on TargetP and PredSL. Al predicted genes are included regardless of their final localization tier, with individual predictor scores ranging from 0 (no localization likelihood) to ~0.9 (high probability). The Top Blast hit ID column corresponds to the top accession match retrieved via homology search using diamond BLASTP against the specialized mitochondrial reference database described by Powers et al. (2022). These alignments were strictly filtered to retain only hits adhering to a threshold of 30% sequence identity (Target ID) and 50% alignment coverage (Target Cover). Target Cover indicates the percentage Target cover to the mitochondrial database for the top Blast hit, and Target ID indicates percentage ID hit to the mitochondrial database for the top Blast hit. Additionally, Eggnog annotation data includes the following metrics: Eggnog E value, COG, Eggnog Description, Preferred Name (when available), EC, Kegg Reaction, and PFAMS.

**Supplementary Data 2.** Nucleotide sequences of predicted genes obtained from MAGs assembled reads, with functional annotations provided in Supplementary Data 1.

**Supplementary Data 3.** Genes identified as electron transport chain (ETC) complex subunits obtained from transcriptome data. Gene IDs (“query”) and annotation details, including EggNOG functional annotations, Preferred_name (when available), and PFAM domain annotations are provided. The corresponding nucleotide and amino acid sequences can be retrieved using the Gene ID (“query”) through the deposited supplementary sequence files and associated identifier mapping files (as noted later in this caption). Rows are color-coded by specific respiratory complex: Complex I (CI; highlighted in orange), Complex II (CII; highlighted in yellow), Complex III (CIII; highlighted in blue), and Complex V (CV; highlighted in green). Unhighlighted entries correspond to the pyrophosphate-energized vacuolar membrane proton pump. Data are accessible via FigShare (<https://doi.org/10.6084/m9.figshare.30287110>) or in the files: gene-call-transcriptome-SBB saccamminid.faa, gene-call-transcriptome-SBB saccamminid.fna, and SBB saccamminid_transcriptome-eggnog.emapper.annotations.

**Supplementary Data 4.** Genes identified as electron transport chain (ETC) complex subunits obtained from metatranscriptome data. Gene IDs (“query”) and annotation details, including EggNOG functional annotations, Preferred_name (when available), and PFAM domain annotations are provided. The corresponding nucleotide and amino acid sequences can be retrieved using the Gene ID (“query”) through the deposited supplementary sequence files and associated identifier mapping files (as noted later in this caption). Rows are color-coded by specific respiratory complex: Complex I (CI; highlighted in orange), Complex II (CII; highlighted in yellow), Complex III (CIII; highlighted in blue), and Complex V (CV; highlighted in green). Unhighlighted entries correspond to the pyrophosphate-energized vacuolar membrane proton pump. Data are accessible via FigShare (<https://doi.org/10.6084/m9.figshare.30287110>), or in the files: gene-calls-metatranscriptome-SBB saccamminid.faa, gene-calls-metatranscriptome-SBB saccamminid.fna, and SBB saccamminid_metatranscriptome-eggnog.emapper.annotations.
